# Use of Peripheral Nerve Blocks with Sedation for Total Knee Arthroplasty in a Patient with Contraindication for General Anesthesia

**DOI:** 10.1155/2015/950872

**Published:** 2015-10-26

**Authors:** Eric Kamenetsky, Antoun Nader, Mark C. Kendall

**Affiliations:** ^1^McGaw Medical Center of Northwestern University, Chicago, IL 60611, USA; ^2^Feinberg School of Medicine, Northwestern University, Chicago, IL 60611, USA

## Abstract

Although peripheral nerve blocks are commonly used to provide postoperative analgesia after total knee arthroplasty (TKA) and other lower extremity procedures, these blocks are rarely used for intraoperative anesthesia. Most TKAs are performed under general anesthesia (GA) or neuraxial anesthesia (NA). The knee has a complex sensory innervation that makes surgical anesthesia difficult with peripheral nerve blocks alone. Rarely are both GA and NA relatively contraindicated and alternatives are considered. We present a patient who underwent TKA performed under peripheral nerve block and sedation alone.

## 1. Introduction

Although peripheral nerve blocks are commonly used to provide postoperative analgesia after total knee arthroplasty (TKA) and other lower extremity procedures, these blocks are rarely used as the sole anesthetic. The majority of TKAs are performed under general anesthesia (GA) in the United States (57.9%), with most other cases utilizing neuraxial anesthesia (NA) [1]. The knee has a complex sensory innervation that makes surgical anesthesia difficult with peripheral nerve blocks (PNBs) alone. The knee is innervated by the lumbosacral plexus. Few publications exist about lumbar plexus block in combination with sciatic nerve block.

Innervation of the anterior knee joint is primarily supplied by the femoral nerve. The posterior knee joint is supplied by the genicular branches of the sciatic nerve, with contribution from the obturator nerve supplying the medial portion of the knee joint. The cutaneous innervation of the thigh and knee involves multiple nerve distributions [2]. The lateral femoral cutaneous nerve (LFCN) supplies portions of the anterior and lateral thigh, extending to the knee. The posterior femoral cutaneous nerve (PFCN) is a pure sensory nerve that, in a majority of cases, emerges from the greater sciatic foramen beneath the piriformis muscle. The PFCN then travels medial to the sciatic nerve and lies directly on the deep surface of the gluteus maximus muscle. Here, the PFCN has been described to divide into many branches which continue inferiorly, supplying sensation to the posterior thigh, popliteal region, and calf area and occasionally extending to the calcaneal region. The anterior femoral cutaneous nerves (AFCNs), which include the intermediate femoral and medial femoral cutaneous nerves, are reported to be branches of the anterior division of the femoral nerve. The intermediate femoral cutaneous nerve supplies the anterior thigh as far distal as the knee, with the medical cutaneous branches supplying the adjacent medial portions of the thigh, not covered by the obturator nerve. Collectively, the sensory nerve branches that terminate at the knee are commonly referred to as the patellar plexus [3, 4].

At our institution, neuraxial blocks are performed as the primary anesthetic for more than 95 percent of TKAs. For the vast majority of cases where NA is contraindicated, a general anesthetic is performed. It is uncommon that both GA and NA are contraindicated and alternatives should be considered. We present a patient who underwent TKA using multiple PNBs with local anesthetic and sedation alone. Written consent for publication was obtained from the patient.

## 2. Case Description

A sixty-one-year old Caucasian female is scheduled for a left TKA. The patient had a history of pulmonary fibrosis requiring 4 L supplemental O_2_ with exercise, OSA, idiopathic thrombocytopenic purpura, gastroesophageal reflux disease, and obesity. She also had a recent history of pulmonary embolism (PE) necessitating early, postoperative anticoagulation for deep venous thrombosis and PE prevention. Preoperative platelet count was 88,000/*μ*L, which was stable for the preceding two years. During a formal six-minute walk test, the patient desaturated to 85% on room air and required 5 L O_2_ to keep SpO_2_ > 88%. Decision was made to proceed with TKA under PNBs and minimal sedation. Complete surgical anesthesia was achieved and patient underwent TKA without complications.

## 3. Block Descriptions

**Figure 1:**
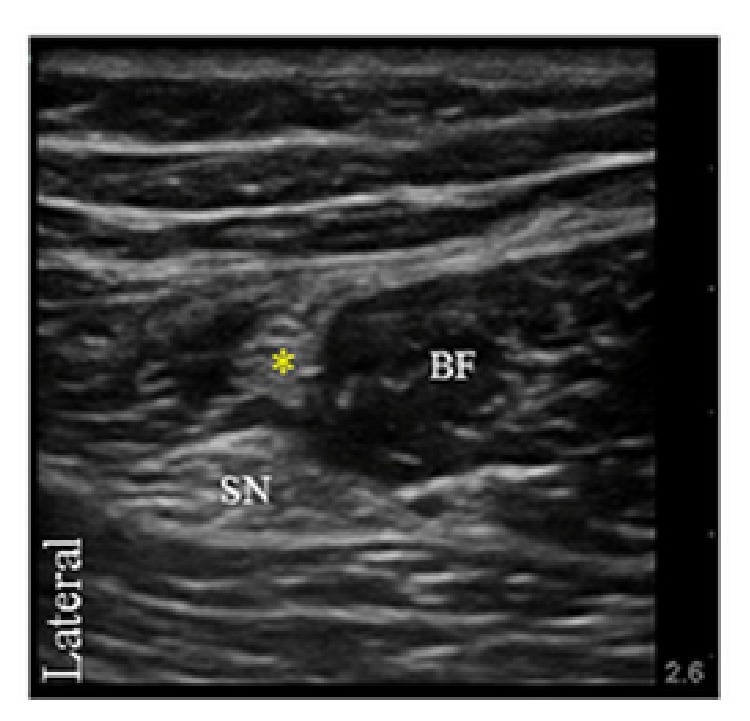
Transverse ultrasound image of the posterior femoral cutaneous nerve (asterisk) located deep to the gluteus maximus muscle and lateral to the biceps femoris muscle (BF). Sciatic nerve (SN).

An ultrasound-guided infragluteal-parabiceps approach was used for sciatic nerve blockade (SNB) [5]. Ten mL of 0.5% bupivacaine with 1 : 300,000 epinephrine was administered deep to the common investing extraneural layer around the sciatic nerve (Figure 1).

**Figure 2:**
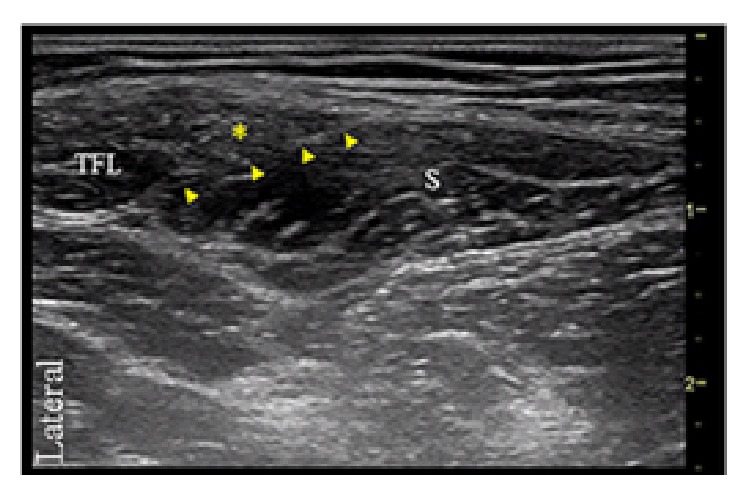
Transverse ultrasound image of the lateral femoral cutaneous nerve (asterisk) lying below fascia lata and lateral to the sartorius muscle (S) and medial to the tensor fasciae latae muscle (TFL). Arrow heads point to the fascia that separates the sartorius muscle from the LFCN.

The PFCN was identified deep to the gluteus maximus muscle, lateral to the biceps femoris, and posterior to the sciatic nerve. Here, 3 mL of 0.5% bupivacaine with 1 : 300,000 epinephrine was injected (Figure 1). With the patient supine, the LFCN was identified lateral to the sartorius muscle, anterior to the iliotibial tract, and below fascia lata. Five mL of 0.5% bupivacaine with 1 : 300,000 epinephrine was administered at this location (Figure 2).

**Figure 3:**
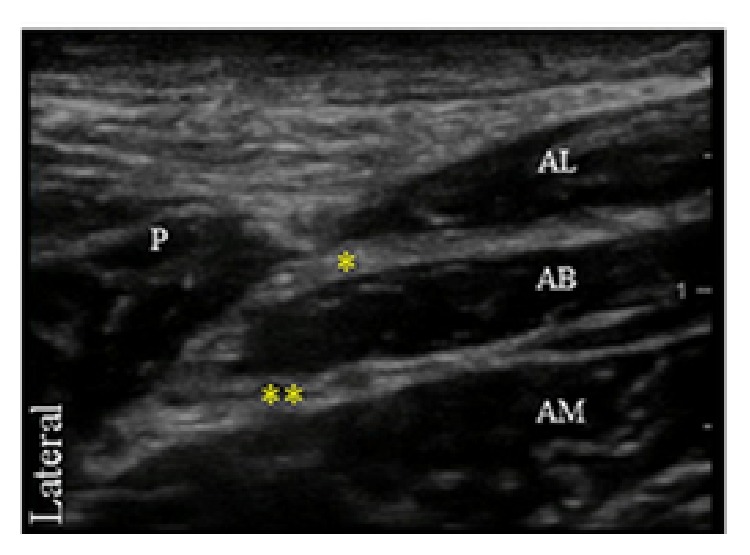
Transverse ultrasound image of the obturator nerve. The distal portion of the obturator nerve divides into an anterior division (single asterisk), identified between the adductor longus (AL) and brevis muscles (AB) and a posterior division (double asterisks) located between adductor brevis and magnus (AM) muscles. Pectineus muscle (P).

The distal divisions of the obturator nerve were identified between the adductor longus and brevis muscles (anterior division) and between adductor brevis and magnus (posterior division). A total of 10 mL of 0.5% bupivacaine with 1 : 300,000 epinephrine was used for the obturator nerve block, 5 mL for each division (Figure 3).

**Figure 4:**
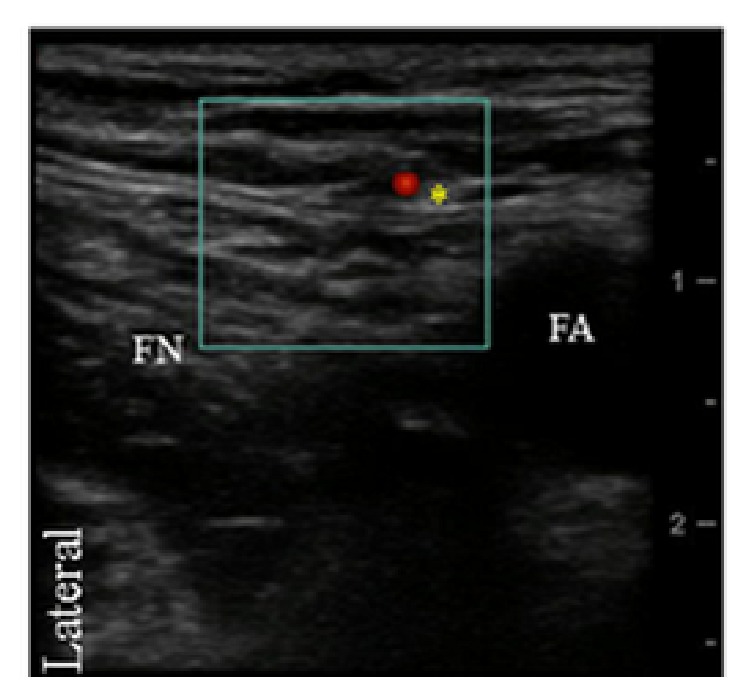
Transverse ultrasound image of the femoral nerve (FN) and femoral artery (FA). The anterior femoral cutaneous nerve of the thigh with its accompanying artery is also shown (asterisk).

The anterior femoral cutaneous nerve was identified at the level of the inguinal crease in a plane superficial to fascia lata and adjacent to a superficial arterial branch of the femoral artery. Here, 2 mL of 0.5% bupivacaine with 1 : 300,000 epinephrine was administered (Figure 4).

Finally, a femoral nerve catheter (FNC) was placed using a sterile technique. A 4 cm 18-gauge stimulating Tuohy needle, connected to the negative lead of a constant voltage nerve stimulator (Stimuplex DIG; B-Braun/McGaw Medical, Bethlehem, PA), was inserted at the inguinal crease using ultrasound guidance (Figure 4). With quadriceps evoked motor response (EMR) at 0.5 mA, a 20 G stimulating catheter (StimuCath; Arrow International, Reading, PA, USA) was advanced while maintaining the evoked motor response of the quadriceps muscle throughout catheter advancement. The femoral catheter was advanced 11 cm beyond the needle tip. A total of 15 mL of 0.5% ropivacaine with 1 : 300,000 epinephrine was administered through the catheter after placement. A 2.5 cm broadband linear array transducer (7–13 MHz probe, Sonosite, M Turbo, Bothell, WA, USA) was used for all PNBs. For the single-shot PNBs, a 21 G echogenic stimulating needle was used (Pajunk, Medizintechnologie, Geisingen, Germany).

## 4. Discussion

Over the last decade, various anesthetic techniques have been used to care for patients undergoing TKA. Regional and neuraxial techniques are often chosen to improve postoperative analgesia and to minimize adverse effects from systemic opioids.

There are several reports of lower extremity surgery performed utilizing a lumbar plexus block (LPB) in combination with a sciatic nerve block. Luber et al. evaluated the efficacy of lumbar plexus block techniques for TKA [6]. Although no complications related to the LBP were reported in this study group of patients, there are several known risks. Retroperitoneal hematomas and other hemorrhagic complications have been reported in case studies [7, 8]. American Society of Regional Anesthesia (ASRA) guidelines state, for patients undergoing deep plexus block, recommendations for neuraxial techniques should be similarly applied to minimize the risk of hemorrhagic complications [9].

Regional techniques specifically targeting the intermediate cutaneous nerve of the thigh with ultrasound guidance are not well described in the literature. A cadaveric study evaluating the fascicular anatomy of the femoral nerve showed that the distance from the inguinal ligament to the first branching point of the femoral nerve was 1.50 ± 0.47 cm [10]. Anterior and medial thigh cutaneous coverage may be partially missed if a FNB is performed distal to its cutaneous branching points, thus resulting in inadequate cutaneous anesthesia of the thigh and knee.

It has been shown that PFCN blockade during SNB is likely consequence of local anesthetic diffusion [11, 12]. A targeted block of the PFCN was performed in our case to ensure anesthesia of the posterior thigh for a thigh tourniquet and to provide cutaneous anesthesia of the posterior knee in an effort to minimize patient appreciation of knee manipulation during TKA.

Using peripheral nerve blocks alone to perform TKA may have several limitations and considerations. First, the total dose of local anesthetic used in this case approaches the maximum recommended dose of local anesthetic. We chose to use ropivacaine when dosing through the FNC for this reason. Also, the technique we describe requires multiple separate needle entry points and perineural injections. Transient postoperative neurologic symptoms after PNBs have been reported to occur at rates from 0 to 15%, with a rate of ~8% seen after nerve-stimulation assisted sciatic nerve blockade [13, 14]. No studies have evaluated the incidence of neurologic sequelae after performing femoral, sciatic, and multiple cutaneous PNBs, similar to the technique used in this case. The innervation of the knee is highly variable, particularly cutaneous innervation, which makes complete surgical anesthesia a challenge using PNBs alone. The techniques we describe achieved complete anesthesia of the knee joint, as well as cutaneous anesthesia. Any small, unidentified gaps in cutaneous coverage were likely compensated by small doses of sedation given intraoperatively.

The techniques we describe may help other anesthesiologists when encountering patients with contraindications for both GA and NA. With the growing demand for TKA, anesthesiologists will likely encounter an increasing number of patients similar to those presented in this case [15].

## References

[1] Fleischut P. M., Eskreis-Winkler J. M., Gaber-Baylis L. K. (2015). Variability in anesthetic care for total knee arthroplasty: an analysis from the anesthesia quality institute. *American Journal of Medical Quality*.

[2] Wasielewski R. C., Callaghan J. J., Rosenberg A. G., Rubash H. E., Simonian P. T., Wickiewicz T. L. (2003). Surgical anatomy of the knee. *The Adult Knee*.

[3] Gray H., Clemente C. D. (1985). *Anatomy of the Human Body*.

[4] Netter F. H. (2006). *Atlas of Human Anatomy*.

[5] Nader A., Kendall M. C., De Oliveira G. S. (2013). A dose-ranging study of 0.5% bupivacaine or ropivacaine on the success and duration of the ultrasound-guided, nerve-stimulator-assisted sciatic nerve block: a double-blind, randomized clinical trial. *Regional Anesthesia and Pain Medicine*.

[6] Luber M. J., Greengrass R., Vail T. P. (2001). Patient satisfaction and effectiveness of lumbar plexus and sciatic nerve block for total knee arthroplasty. *The Journal of Arthroplasty*.

[7] Weller R. S., Gerancher J. C., Crews J. C., Wade K. L. (2003). Extensive retroperitoneal hematoma without neurologic deficit in two patients who underwent lumbar plexus block and were later anticoagulated. *Anesthesiology*.

[8] Aveline C., Bonnet F. (2004). Delayed retroperitoneal haematoma after failed lumbar plexus block. *British Journal of Anaesthesia*.

[9] Horlocker T. T., Wedel D. J., Rowlingson J. C. (2010). Regional anesthesia in the patient receiving antithrombotic or thrombolytic therapy: American Society of Regional Anesthesia and Pain Medicine Evidence-Based Guidelines (third edition). *Regional Anesthesia & Pain Medicine*.

[10] Gustafson K. J., Pinault G. C. J., Neville J. J. (2009). Fascicular anatomy of human femoral nerve: implications for neural prostheses using nerve cuff electrodes. *Journal of Rehabilitation Research and Development*.

[11] Barbero C., Fuzier R., Samii K. (2004). Anterior approach to the sciatic nerve block: adaptation to the patient's height. *Anesthesia and Analgesia*.

[12] Chelly J. E., Delaunay L. (1999). A new anterior approach to the sciatic nerve block. *Anesthesiology*.

[13] Brull R., McCartney C. J. L., Chan V. W. S., El-Beheiry H. (2007). Neurological complications after regional anesthesia: contemporary estimates of risk. *Anesthesia and Analgesia*.

[14] Nader A., Kendall M. C., Doty R. (2011). Nerve stimulator-guided supplemental popliteal sciatic nerve block after a failed sciatic block does not increase the incidence of transient postoperative neurologic sequelae. *Anesthesiology*.

[15] Kurtz S., Ong K., Lau E., Mowat F., Halpern M. (2007). Projections of primary and revision hip and knee arthroplasty in the United States from 2005 to 2030. *Journal of Bone and Joint Surgery A*.

